# The safety and efficacy of prednisolone in preventing reaccumulation of ascites among endomyocardial fibrosis patients in Uganda: a randomized clinical trial

**DOI:** 10.1186/s13104-015-1761-0

**Published:** 2015-12-15

**Authors:** Yvonne Brenda Nabunnya, James Kayima, Chris T. Longenecker, Richard A. Josephson, Juergen Freers

**Affiliations:** Department of Medicine, Makerere University College of Health Sciences, P.O. Box 7072, Kampala, Uganda; Division of Cardiovascular Medicine, Case Western Reserve University School of Medicine, Cleveland, USA; University Hospitals Case Medical Center, Harrington Heart and Vascular Institute, Cleveland, USA

**Keywords:** Prednisolone, Endomyocardial fibrosis, Ascites

## Abstract

**Background:**

Endomyocardial fibrosis (EMF), the commonest restrictive cardiomyopathy worldwide, is characterized by inflammation and fibrosis of the endocardium. Inflammation in other parts of the body such as the peritoneum has been described and may explain the accumulation of ascites, a painful and disabling feature of this disease. We determined the efficacy and safety of prednisolone to prevent re-accumulation of ascites among EMF patients attending Mulago hospital cardiology service.

**Methods:**

This was a pilot randomised placebo controlled trial with a 1:1 parallel design. Over a period of 10 months, participants were recruited and randomized to receive 1 mg/kg per day of prednisolone or placebo and were followed for a maximum of 8 weeks. The primary outcome was re-accumulation of ascites from baseline (grade 2) to grade 3. Safety was assessed by self-reported side effects, physical exam, and laboratory assessment.

**Results:**

Sixteen study participants were randomised to prednisolone, while nineteen were randomised to placebo. Six were lost to follow up (1-prednisolone arm, 5-placebo). Baseline characteristics were similar between groups. Prednisolone was safely administered in this setting. There was no statistically significant difference in the overall risk of developing grade 3 ascites over 8 weeks. RR (95 % confidence interval) 0.70 (0.43–1.11), P value 0.12. The rate of the primary outcome per 1000 person days of follow-up was also similar in both arms P value 0.63.

**Conclusion:**

Short term prednisolone use was generally safe and did not prevent reaccumulation of ascites in this study population.

Trial registration number: ISRCTN63999319, 28/03/2013

## Background

Endomyocardial fibrosis (EMF) is the commonest restrictive cardiomyopathy worldwide . It was first recognized during the 1940s and is still a cause of heart failure in Uganda [1]. A recent community-based study of over 1000 people in Mozambique found an overall prevalence of 19.8 %. The commonest observed presentation of this disease is ascites which often requires repeated paracentesis because current medical treatment which includes diuretics, digoxin and angiotensin converting enzyme inhibitors does not spare the patient from repeated paracenteses [2].

While data on the management of ascites due to EMF are lacking, there is a state of clinical equipoise amongst physicians managing these patients on the usefulness of corticosteroid therapy in preventing accumulation of ascites. The use of corticosteroids in the management of EMF, although not backed by randomised clinical trials, is informed by the pathological finding of global inflammation and fibrosis which is not limited to the heart. The infiltration of the peritoneal cavity with leukocytes and exudative ascites suggests on-going inflammation of the peritoneum that is independent of disease duration [2]. It is therefore plausible that anti-inflammatory therapy can be useful to slow the progression of disease [2–6]. Indeed, anecdotal reports in African settings have described complete disappearance of ascites with corticosteroids therapy; however, safety, efficacy, and optimal duration of therapy of steroid use are all unknown. Prednisolone is an intermediate acting corticosteroid drug with predominant glucocorticoid and low mineral corticoid activity, making it useful for the treatment of a wide range of inflammatory and auto-immune conditions. It is well absorbed from the gastrointestinal tract, and widely distributed throughout the body plasma protein bound on transcortin and albumin. It was chosen because it is inexpensive and readily available in Uganda. It has a moderate side effect profile and the pill burden is limited compared to other corticosteroids such as dexamethasone.

In this patient population, abdominal swelling due to ascites is the commonest presenting feature of EMF, and is often accompanied by abdominal pain, general weakness, and effort intolerance. The ascites is usually out of proportion to the amount of peripheral oedema [2]. Recurrent hospitalisation and paracenteses result in high social and economic costs to patients and caretakers. In this pilot study, we determined the safety and efficacy of prednisolone to prevent re-accumulation of ascites among EMF patients in Uganda.

## Methods

This was a double-blind, randomised, placebo-controlled trial of patients with endomyocardial fibrosis and ascites conducted at Mulago National Referral Hospital (Kampala, Uganda) from April 2012 to January 2013. Scientific and ethical approval was obtained from Makerere University College of Health Sciences School of Medicine Research and Ethics Committee (REC REF 2011-252), The National Drug Authority, and the trial registration number ISRCTN63999319 was obtained from www.controlled-trials.com. We hypothesised that a sample of 19 participants per group would give the study 80 % power to detect a 35 % difference between the two groups. This was based on the assumption that 95 % of patients would develop grade 3 ascites with standard care [2, 4]. Ascites was graded as follows using the International Ascites Club (IAC) grading grade 1: mild, only visible on ultrasound, grade 2: moderate symmetrical distension of abdomen and grade 3: large or gross ascites with marked abdominal distension [7]. Starting with all study participants at IAC grade 2, the primary end point for the efficacy outcome was re-accumulation to IAC grade 3.

Participants were recruited from the outpatient cardiology clinic and inpatient ward Mulago Hospital Complex with the assistance of two research assistants and a screening questionnaire. Inclusion criteria were (a) 13 years and older and (b) a diagnosis of EMF with ascites based on published echocardiographic criteria [8]. Participants were excluded if they were critically ill, had unstable vital parameters, or were pregnant. Those previously exposed to corticosteroids were allowed to enrol after a wash out period of 1 month. Written informed consent was obtained from all participants over 18 years of age. Assent and parental consent were obtained from participants younger than 18 years. A data safety monitoring board (DSMB) was responsible for the review the unblinded data.

### Study procedures

Baseline demographics and medical history were obtained using a standard questionnaire. A physical exam was performed to measure abdominal girth, weight, and grade of ascites. All participants with international ascites grade 3 ascites underwent paracentesis so that no participant had greater than international ascites grade 2 ascites at baseline. Leukocyte count and albumin were measured in all samples of ascites. Venous blood was collected for a complete blood count with differential, blood glucose, and albumin. The serum-to-ascites albumin gradient (SAAG) was calculated as the difference between the ascites and serum albumin concentrations.

Participants were randomized to the prednisolone or placebo groups using a computer-generated sequence of random numbers generated by an epidemiologist. Blinding was done by using tablets of prednisolone and placebo with similar shape, size and taste from the manufacturer. The drugs were packaged by the manufacturer who did not know the study hypothesis into opaque tins. A sealed envelope containing the actual drug codes was kept by the study epidemiologist. The principal investigator assigned participants to a specific study arm while the research assistants dispensed the drugs to the patients who did not know to which study arm they had been assigned. The seal on the drug codes was only broken at the end after the final analysis had been done.

Prednisolone was administered orally at a dose of 1 mg/kg per day or matching placebo at the same dose with a ceiling dose of 60 mg per day. All study participants were followed up every 4 weeks until they reached the primary endpoint or for a maximum of 8 weeks. When the primary end point was reached, the study drugs were tapered off by halving the dose every week over a period of 1 month and then stopped. They were then redirected to the cardiac clinic for regular follow up and care. At each follow-up visit, side-effects were assessed by self-report using a standardized questionnaire and a physical exam. A random blood sugar was done at each visit using a contour glucometer. Adherence to treatment was assessed by pill count.

### Statistical analysis

All analyses were performed using STATA software package, version 11 and it was on intention to treat basis. Baseline characteristics of study participants were described as mean (standard deviation) for continuous variables or number (percent) for categorical variables, and were compared using t-tests, Wilcoxon Rank Sum tests, Chi-square tests, or Fisher’s Exact tests as distributionally appropriate.

The primary outcome of this study was accumulation of grade 3 ascites requiring paracentesis. The relative risk of the primary outcome in the prednisolone group was calculated as the ratio of the proportion of participants who reached grade 3 ascites in the prednisolone group and the placebo group. In a separate analysis, the time to reaccumulation of grade 3 ascites was compared between groups. The frequencies of specific adverse events were described by study group and were compared using Chi-square tests or Fisher’s Exact tests as distributionally appropriate. All tests were two-sided and a P value of <0.05 was considered statistically significant.

## Results

**Fig. 1:**
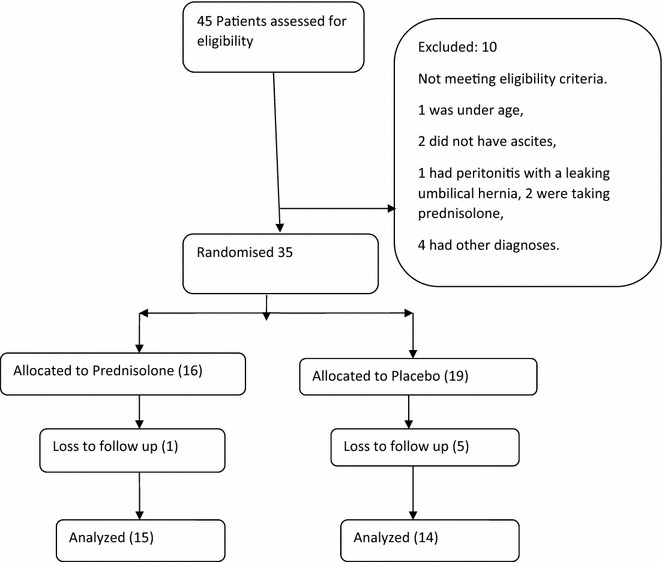
Participant flow chart. Showing the number of patients screened, recruited, randomized, allocated to the prednisolone or placebo groups, and analyzed

**Fig. 2:**
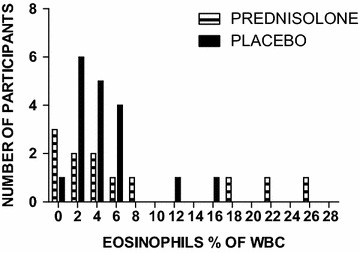
Histogram of percentage eosinophil distribution among participants on prednisolone and placebo arms, showing only 10 % of participants having eosinophilia

Study flow chart is described in Fig. 1. All study participants received the study drug according to their allocated study arm. Overall adherence to treatment was 81 % and did not differ between groups P value 0.77. Baseline clinical characteristics of study participants were similar in both groups (Table 1). Significant baseline laboratory characteristics include the following: peripheral eosinophilia (>10 % of total leukocytes) was observed in only 10 % of patients (Fig. 2), majority (97 %) had a transudative ascites, with 80 % having a lymphocytic predominance of cells on ascitic fluid analysis.

**Table 1:** Baseline demographic characteristics of study participants

Characteristic	Prednisolone, n (%)	Placebo, n (%)	P value
Mean age	27.947 (SD 9.50)	27.125 (SD 8.82)	0.709
Sex			1.000
Male	5 (36.84)	7 (31.25)	
Female	11 (68.75)	12 (63.16)	
Level of education			0.491
No formal education	2 (12.50)	4 (21.05)	
Primary	12 (75.00)	10 (52.63)	
Secondary	2 (12.50)	5 (26.32)	
Occupation			0.636
Unemployed	11 (68.75)	13 (68.42)	
Employed	5 (31.25)	6 (31.58)	
Duration of disease			0.529
<1 year	13 (81.25)	11 (57.89)	
1–2 years	1 (6.25)	1 (5.26)	
3–5 years	1 (6.25)	3 (15.79)	
>5 years	1 (6.25)	4 (21.05)	
Family history of EMF			0.398
Yes	4 (25.00)	3 (15.795)	
No	12 (75.00)	16 (84.21)	
Paracentesis in prior 3 months			0.763
None	6 (37.50)	8 (42.11)	
Once	1 (6.25)	3 (15.79)	
Twice	4 (25.00)	4 (21.05)	
Thrice	3 (18.75)	1 (5.26)	
More than 3 times	2 (12.50)	3 (15.79)	
Mean weight	47.894 (SD 10.32)	45.625 (SD 15.56)	0.703
Blood pressure			0.234
<90/60	0 (0.00)	3 (15.79)	
90/60–139/89	16 (100.00)	16 (84.21)	
Temperature			0.457
<35	1 (6.25)	0 (0.00)	
35–37.5	15 (93.75)	19 (100.00)	
Random blood sugar			0.481
<4 mmol/dl	4 (25)	6 (31.58)	
4–11 mmol/dl	12 (75.00)	13 (68.42)	
White cell count (WBC)			0.758
>10,000	1 (8.33)	1 (5.88)	
4000–10,000	7 (58.33)	8 (47.06)	
<4000	4 (33.33)	8 (47.06)	
Eosinophilia (>10 % WBC)	2 (16.67)	1 (5.88)	0.348
Haemoglobin			0.793
>14 mg/dl	9 (75.00)	12 (70.59)	
12–14 mg/dl	3 (25.00)	5 (29.41)	
SAAG			0.414
<1.1	1 (8.33)	0 (0.00)	
>1.1	11 (91.67)	17 (100)	
Ascitic fluid lymphocytes			0.091
>40 %	15 (93.75)	13 (68.42)	
<40 %	1 (6.25)	6 (31.58)

### Primary outcome

**Fig. 3:**
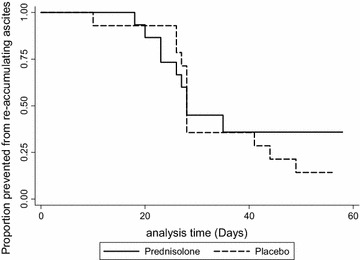
Kaplan Meier curve comparing the proportion of patients prevented from re-accumulating ascites in the prednisolone and placebo arms. There was no statistically significant difference in the rate of re-accumulation between the groups (P = 0.63)

The mean follow-up was 34 days in the prednisolone arm and 35 days in the placebo arm. Of the 29 patients who completed longitudinal follow-up, 21 reached the primary endpoint [n = 9 (60 %) vs n = 12 (86 %) for prednisolone vs placebo, respectively; relative risk (95 % confidence interval, CI) 0.7 (0.439–1.114), P = 0.12]. The rate of the primary outcome per 1000 person days of follow up in the prednisolone group was 17.6 (95 % CI 9.1–33.8) vs 25.6 (14.6–45.1) in the placebo group (P = 0.63) (Fig. 3).

### Adverse events

All adverse events were reviewed by the DSMB. There were three serious (>grade 3) adverse events over the course of the study. One patient in the prednisone group was hospitalized for malaria, and was discharged after 3 days. In the placebo group, one was hospitalized for spontaneous bacterial peritonitis, and was discharged after 5 days. One participant in the placebo group died before the first follow-up visit from an acute illness characterized by severe dyspnea as described by family members.

The most common minor (grade 2 or less) adverse event was epigastric pain (73 %), followed by muscle pain and melena (Table 2). No study participant had a significant drop in haemoglobin or required a blood transfusion from gastrointestinal bleeding. There was one patient in the prednisolone group who developed diabetes with a random blood sugar of 24.5 mmol/dl. For this patient, the prednisolone was tapered off and the random blood sugar fell to 17.5 mmol/dl and she was referred for further care.

**Table 2:** Adverse events of study participants

Adverse event	Prednisolone, n (%)	Placebo, n (%)	P value
Epigastric pain	11 (73.33)	7 (50.00)	0.201
Malena	1 (6.67)	3 (21.43)	0.328
Muscle pain	4 (26.67)	2 (14.29)	0.417
Blood pressure			0.273
<90/60	1 (6.67)	3 (21.43)	
90/60–139/89	14 (93.33)	11 (78.57)	
Random blood sugar			
<4 mmol/dl	4 (26.67)	5 (35.71)	0.685
7–11 mmol/dl	3 (60)	2 (40)	0.361
>11 mmol/dl	1 (6.67)	0 (0.00)	0.723

## Discussion

In this clinical trial of short-term prednisolone use in symptomatic EMF with ascites, we found that prednisolone does not appear to reduce the re-accumulation of ascites.

That prednisolone did not reduce reaccumulation of ascites can be explained by the fact that we found little evidence of ongoing peritoneal inflammation (less eosinophilia and predominantly transudative ascites) contrary to earlier studies [12].

To our knowledge, this is the first clinical trial of anti-inflammatory treatment for EMF. The baseline characteristics of our subjects were mostly in agreement with what is known about the risk factors for this disease [9]. The differences, of a small number of our study population having eosinophilia and majority having transudative ascites could have been because we saw these patients late after the acute stage of disease had passed [10], or it could be that the picture of EMF in our context is different than the patients described by Rutakingirwa et al. [11].

In this study population, there was no significant difference in the occurrence of adverse effects among the two study arms. There was a number of self-limited gastrointestinal bleeds but none requiring blood transfusion [13]. Prednisolone increased the risk of developing epigastric pain and muscle pains by 23 and 12 % respectively, but this was not statistically significant between groups. Only one study participant developed high random blood sugar which reduced on stopping the study drug. The short duration of drug exposure was the main study limitation.

## Conclusion

Prednisolone did not prevent reaccumulation of ascites in this patient population but was safe. Exploratory studies in the aetiology and pathophysiology of this disease may throw more light and guide therapeutic attempts among such patients.
